# Activation of Astroglial Connexin Is Involved in Concentration-Dependent Double-Edged Sword Clinical Action of Clozapine

**DOI:** 10.3390/cells9020414

**Published:** 2020-02-11

**Authors:** Kouji Fukuyama, Ruri Okubo, Masahiko Murata, Takashi Shiroyama, Motohiro Okada

**Affiliations:** 1 Department of Neuropsychiatry, Division of Neuroscience, Graduate School of Medicine, Mie University, Tsu 514-8507, Japan; mk_psy_isui@hotmail.com (K.F.); ddduck0602@gmail.com (R.O.); takashi@clin.medic.mie-u.ac.jp (T.S.); 2 National Hospital Organization Sakakibara Hospital, 777 Sakakibara, Tsu, Mie 514-1292, Japan; muyuhton@gmail.com

**Keywords:** clozapine, schizophrenia, hemichannel, connexin

## Abstract

Clozapine (CLZ) is a gold-standard antipsychotic against treatment-refractory schizophrenia, but is one of the most toxic antipsychotic agents. Pharmacological mechanisms of the double-edged sword clinical action of CLZ remain to be clarified. To explore the mechanisms of CLZ, the present study determined the astroglial transmission associated with connexin43 (Cx43), which is the most principal expression in astrocytes and myocardial cells, and expression of Cx43 in primary cultured astrocytes. Both acute and subchronic administrations of CLZ concentration-dependently increased Cx43-associated astroglial release of l-glutamate and d-serine, whereas therapeutic-relevant concentration of CLZ acutely did not affect but subchronically increased astroglial release. In contrast, after the subchronic administration of therapeutic-relevant concentration of valproate (VPA), acute administration of therapeutic-relevant concentration of CLZ drastically increased Cx43-associated astroglial releases. VPA increased Cx43 expression in cytosol fraction without affecting plasma membrane fraction, whereas CLZ increased Cx43 expression in both fractions. Acute administration of therapeutic-relevant concentration of CLZ drastically increased Cx43 expression in the plasma membrane fraction of astrocytes subchronically treated with VPA. The present findings suggest that CLZ-induced the activation of Cx43-associated channel activity and transported Cx43 to plasma membrane, probably contribute to the double-edged sword clinical action of CLZ, such as improvement of cognitive dysfunction and CLZ-induced myocarditis.

## 1. Introduction

It has been established that dysfunctions of both dopaminergic and glutamatergic transmission play important roles in the pathophysiology of schizophrenia, with various antipsychotics improving dysfunctions of mesolimbic and mesocortical dopaminergic transmissions with thalamocortical glutamatergic transmission [1,2,3,4,5,6,7]; however, schizophrenia is considered to be a heterogeneous disorder that is unlikely to be caused by a single etiological factor, but rather by a complex network of interacting pathogenic influences [8,9]. Impairment of the *N*-methyl-d-aspartate (NMDA)/glutamate receptor (NMDAR) contributes to the pathophysiology of schizophrenia [1,2,3,6,9,10,11,12]. Several clinical reports have demonstrated that NMDAR antagonists (e.g., phencyclidine and ketamine) can generate schizophrenia-like positive and negative symptoms in healthy volunteers [1,13,14] and exacerbate psychosis in patients with schizophrenia [15]. Moreover, NMDAR antagonist-induced psychosis models exhibit features of schizophrenia, such as negative symptoms and cognitive deficits, more closely than the amphetamine/dopamine psychosis models [16]. Based on clinical and preclinical evidence, dysfunctional glutamatergic transmission associated with NMDAR seems to produce a schizophrenia-like state.

Clozapine (CLZ) is considered the double-edged sword antipsychotic drug, since CLZ is one of the most effective but toxic antipsychotics against antipsychotics-resistant schizophrenia [17]. In fact, despite causing myocarditis, cardiomyopathy, agranulocytosis, and seizures [18,19,20,21,22], CLZ remains the gold-standard antipsychotic for treatment-refractory schizophrenia and is the only approved medication licensed for this indication [17,23]. The mechanisms of clinical actions of CLZ against antipsychotics-resistant schizophrenia have been considered to be modulated by multimodal targets actions, including NMDAR and dopamine D2 receptor. An effect on NMDAR function may be involved in the antipsychotic efficacy of CLZ [6,24], based on evidence from a double-blind, placebo-controlled clinical study in which CLZ significantly blunted a ketamine-induced increase in positive symptoms [25]. Indeed, the astroglial releases of l-glutamate and d-serine induced by CLZ are one of the major mechanisms of agonistic action of CLZ against impaired NMDAR [24].

In terms of management of CLZ-induced seizure, possible strategies include reduction of CLZ dose and addition of antiepileptic drugs, including valproate (VPA), which is the most commonly used for management of CLZ-induced seizures [18,21] due to its additional effect as a mood stabilizer, minimal effect on metabolism of CLZ, and reduction in generalized poly-spikes and wave electroencephalogram pattern in patients on CLZ [20]. Contrary to CLZ-induced seizure, during CLZ commencement, rapid CLZ titration and VPA intake are factors that increase the risk of CLZ-induced myocarditis/cardiomyopathy [22].

Both clinical and preclinical studies have emphasized that thalamocortical disturbance is particularly relevant for cognitive dysfunction in several neuropsychiatric disorders, including schizophrenia, Attention-deficit hyperactivity disorder (ADHD), intellectual disability, autism, and epileptic psychosis [3,4,5,6,7,9,26,27]. Recently, we demonstrated that thalamocortical glutamatergic transmission from mediodorsal thalamic nucleus (MDTN) to medial prefrontal cortex (mPFC) was activated by attenuation of intrathalamic GABAergic transmission from reticular thalamic nuclei (RTN) to MDTN [3,4,5,9,26,27], whereas therapeutic-relevant concentration of CLZ compensates hyperfunction of thalamocortical glutamatergic transmission via activation of frontal group III metabotropic glutamate receptors [6].

Accumulating evidences indicate connexin (Cx) composed transmembrane channels (Cxs) are crucial to the coordination and maintenance of physiologic activity including neuronal excitability, synaptic plasticity, tripartite synaptic transmission, and homeostasis maintenance in the central nervous system [28,29]. Cx is a family of 21 protein isoforms, and 11 Cx isoforms are expressed in the central nervous system [28,29,30]. Six Cx proteins assemble to form homomeric or heteromeric connexons. Two connexons in two neighboring cells (including neuron, astrocyte, oligodendrocyte, and microglia) form a gap junction channel with an aqueous pore and charged surface walls [28,29,30]. Single connexon contributes to chemical connection between intra- and extracellular spaces as hemichannel [28,29,30]. However, pathological conditions, including ischemia and excessive depolarization, generate persistent Cxs-associated hemichannel/gap junction opening, which leads to the disruption of several homeostasis systems [28,29]. Connexin43 (Cx43) is the most widely expressed Cx subtype and most predominant component in the astroglial and myocardial gap-junction/hemichannel Cxs [31,32]. Upregulation of Cx43 generates prolongation of QRS complex duration in electrocardiogram, which is the risk for morbidity and mortality in various cardiac diseases [33]. Furthermore, the expression of Cx43 is increased in glia but not neuron in animal models and patients with epilepsy [34]. Exactly, inhibitors of Cxs can prevent the onset of epileptic seizures [29,35,36]. Although there have not been studies of Cx43 alterations in brains from subjects with schizophrenia, the dysfunction of Cxs contributes to attenuated information processing and cognitive impairment, which is a major symptom of schizophrenia [8].

Our recent preclinical study demonstrated that CLZ acutely activated astroglial Cxs activity, in a concentration-dependent manner. Clinically, survival analysis has suggested that the lower limit of the therapeutic range of CLZ serum concentration is 0.6 μM [37], whereas exceeding 4 μM significantly increases the risk of seizures [38]. Lower than 3 μM CLZ did not affect the astroglial Cxs, whereas toxic concentrations of CLZ (higher than 10 μM) activated astroglial Cxs activity. These clinical and preclinical findings suggest that the mechanism of clinical action of CLZ is a candidate in the function of Cxs associated with Cx43; however, the effect of chronic administration of CLZ on tripartite synaptic transmission associated with Cxs remains to be clarified. Based on these backgrounds, to clarify the mechanisms of double-edged sword clinical action of CLZ, the present study determined the concentration-dependent effects of acute and subchronic administrations of CLZ and VPA on astroglial releases of l-glutamate and d-serine associated with Cxs and the astroglial expression of Cx43 using primary cultured astrocytes.

## 2. Materials and Methods

### 2.1. Chemical Agents

Clozapine (CLZ) was purchased from Sigma (St. Louis, MO, USA). Sodium valproate (VPA) was obtained from Wako Chemicals (Osaka, Japan). The hemichannel/gap junction (Cxs) blocker, carbenoxolone (CBX) [39], and selective Cx43 inhibitor, TAT-conjugated Gap19 (GAP19) [39], were obtained from Funakoshi (Tokyo, Japan). CBX and GAP19 were dissolved in artificial cerebrospinal fluid (ACSF) directly. VPA was dissolved in ACSF or Dulbecco’s modified Eagle’s medium containing 10% fetal calf serum (fDMEM) directly. CLZ was initially dissolved in 10 mM with 1N HCl, then diluted to 1 mM with ACSF or fDMEM.

### 2.2. Preparation of Primary Astrocyte Culture

All animal care and experimental procedures described in this report complied with the Ethical Guidelines established by the Institutional Animal Care and Use Committee at Mie University, Japan (No. 2019-3-R1) and are reported in accordance with the Animal Research: Reporting of In Vivo Experiments (ARRIVE) guidelines [40]. Astrocytes were prepared using a protocol adapted from previously described methods [5,6,12,24,41,42,43,44].

Pregnant Sprague-Dawley rats (SLC, Sizuoka, Japan), which were housed individually in cages, were kept in air-conditioned rooms (temperature, 22 ± 2 °C) set at 12 h light/dark cycle, with free access to food and water. Cultured astrocytes were prepared from cortical astrocyte cultures of neonatal Sprague-Dawley rats (*N* = 48) sacrificed by decapitation at 0–24 h of age. The cerebral hemispheres were removed under dissecting microscope. Tissue was chopped into fine pieces using scissors and then triturated briefly with micropipette. Suspension was filtered using 70 µm nylon mesh (BD, Franklin Lakes, NJ, USA) and centrifuged. Pellets were then resuspended in 10 mL Dulbecco’s modified Eagle’s medium containing 10% fetal calf serum (fDMEM), which was repeated three times. After culture for 14 days (DIV14), contaminating cells were removed by shaking in standard incubator for 16 h at 200 rpm. On DIV21, astrocytes were removed from flasks by trypsinization and seeded directly onto translucent poly ethylene terephthalate (PET) membrane (1.0 μm) with 24-well plates (BD) at a density of 1 × 105 cells/cm^2^ for experiments From DIV21 to DIV28, the culture medium (fDMEM) was changed twice a week, and CLZ (0, 1, 3, 10, 30, or 100 μM) or VPA (0, 300, 1000, or 3000 μM) was added for subchronic administrations (7 days). On DIV28, cultured astrocytes were washed out using ACSF, and this was repeated three times. The ACSF comprised NaCl 150.0 mM, KCl 3.0 mM, CaCl_2_ 1.4 mM, MgCl_2_ 0.8 mM, and glucose 5.5 mM, buffered to pH 7.3 with 20 mM HEPES buffer. The remaining adherent cells contained 95% GFAP-positive and A2B5-negative cells, detected using immunohistochemical staining [45]. After the wash-out, astrocytes were incubated in ACSF (100 μL translucent PET membrane) containing CLZ (0, 1, 3, 10, 30, or 100 μM) or VPA (0, 300, 1000, or 3000 μM) at 35 °C for 60 min in CO_2_ incubator (pretreatment incubation). After the pretreatment, astrocytes were then incubated in ACSF, 50 mM K^+^ (MK-ACSF: NaCl 103.0 mM, KCl 50.0 mM, CaCl_2_ 1.4 mM, MgCl_2_ 0.8 mM, and glucose 5.5 mM, buffered to pH 7.3 with 20 mM HEPES buffer) or 100 mM K^+^ (HK-ACSF: NaCl 53.0 mM, KCl 100.0 mM, CaCl_2_ 1.4 mM, MgCl_2_ 0.8 mM, and glucose 5.5 mM, buffered to pH 7.3 with 20 mM HEPES buffer) containing the same agents of pretreatment (20 min) and collection of the ACSF, MK-ACSF, or HK-ACSF for analysis. Each 100 μL of collected ACSF, MK-ACSF, or HK-ACSF was filtered by Vivaspin 500-3K (Sartorius, Goerringen, Germany) and freeze-dried for storage at −80 °C until needed for analyses.

After the sampling of astroglial transmitter releases, plasma membrane proteins and cytosol proteins of cultured astrocytes were extracted using Minute Plasma Membrane Protein Isolation Kit (Invent Biotechnologies, Plymouth, MN, USA). Both plasma membrane and cytosol fractions were solubilized by radio immunoprecipitation assay buffer (Fujifilm-Wako, Osaka, Japan) containing protease inhibitor cocktail (Nacalai Tesque, Kyoto, Japan).

### 2.3. Study Designs

#### 2.3.1. Concentration-Dependent Effects of Acute Administration of CLZ on Astroglial Releases of l-Glutamate and D-serine (Study_1)

During DIV21 to DIV28, astrocytes were incubated in fDMEM, not containing any target agents. On DIV28 during pretreatment incubation, astrocytes were incubated in ACSF containing CLZ (0, 1, 3, 10, 30, or 100 μM) for 60 min. After pretreatment incubation, astrocytes were incubated in ACSF, MK-ACSF (50 mM K^+^ containing ACSF), or HK-ACSF (100 mM K^+^ containing ACSF) containing the same agent of pretreatment incubation for 20 min. Especially, to determine the astroglial releases associated with Cxs, after pretreatment incubation, astrocytes were incubated in ACSF, MK-ACSF, or HK-ACSF containing the same agent of pretreatment incubation with Cx43 selective inhibitor, CAP19 (20 μM), or nonselective Cxs inhibitor, CBX (100 μM), for 20 min.

#### 2.3.2. Concentration-Dependent Effects of Subchronic Administration of CLZ on Astroglial Releases of l-glutamate and d-serine (Study_2)

During DIV21 to DIV28, astrocytes were incubated in fDMEM containing CLZ (0, 1, 3, 10, 30, or 100 μM). On DIV28 during pretreatment incubation, astrocytes were incubated in ACSF containing the same agent for 60 min. After pretreatment incubation, astrocytes were incubated in ACSF, MK-ACSF, or HK-ACSF containing the same agent for 20 min. Especially, to determine the astroglial releases associated with Cxs, after pretreatment incubation, astrocytes were incubated in ACSF, MK-ACSF, or HK-ACSF containing the same agent with CAP19 (20 μM) or CBX (100 μM) for 20 min.

#### 2.3.3. Concentration-Dependent Effects of Acute and Subchronic Administration of VPA on Astroglial Releases of l-glutamate and d-serine (Study_3)

To determine the effects of acute administration of VPA on astroglial releases of l-glutamate and d-serine, during DIV21 to DIV28, astrocytes were incubated in fDMEM, not containing any target agents. On DIV28 during pretreatment incubation, astrocytes were incubated in ACSF containing VPA (0, 300, 1000, or 3000 μM) for 60 min. After pretreatment incubation, astrocytes were incubated in ACSF, MK-ACSF, or HK-ACSF containing the same agent of pretreatment incubation for 20 min.

To determine the effects of subchronic administration of VPA on astroglial releases of l-glutamate and d-serine, during DIV21 to DIV28, astrocytes were incubated in fDMEM containing VPA (0, 300, 1000, or 3000 μM). On DIV28 during pretreatment incubation, astrocytes were incubated in ACSF containing the same agent for 60 min. After pretreatment incubation, astrocytes were incubated in ACSF, MK-ACSF, or HK-ACSF containing the same agent of pretreatment incubation for 20 min.

#### 2.3.4. Acute Effects of Therapeutic-Relevant Concentration of VPA on Astroglial Releases of l-glutamate and d-serine from Astrocytes Subchronically Administrated with CLZ (Study_4)

During DIV21 to DIV28, astrocytes were incubated in fDMEM containing CLZ (0, 1, 3, 10, 30, or 100 μM). On DIV28 during pretreatment incubation, astrocytes were incubated in ACSF containing the same agent (CLZ) with therapeutic-relevant concentration of VPA (1000 μM) for 60 min. After pretreatment incubation, astrocytes were incubated in ACSF, MK-ACSF, or HK-ACSF containing the same agent of pretreatment incubation for 20 min.

#### 2.3.5. Acute Effects of CLZ on Astroglial Releases of l-glutamate and d-serine from Astrocytes Subchronically Administrated with VPA (Study_5)

During DIV21 to DIV28, astrocytes were incubated in fDMEM containing the therapeutic-relevant concentration of VPA (1000 μM). On DIV28 during pretreatment incubation, astrocytes were incubated in ACSF containing the same agent (1000 μM VPA) with CLZ (0, 1, 3, 10, 30, or 100 μM) for 60 min. After pretreatment incubation, astrocytes were incubated in ACSF, MK-ACSF, or HK-ACSF containing the same agent for 20 min.

### 2.4. Determination of Levels of l-glutamate and GABA

Levels of L-glutamate and GABA were determined by the fluorescence resonance energy transfer method [46,47,48] using UHPLC (xLC3185PU, Jasco, Tokyo Japan) with a fluorescence detector (xLC3120FP, Jasco) after dual derivatization with isobutyryl-l-cysteine and o-phthalaldehyde [3,4,7,9,26,27]. Derivative reagent solutions were prepared by dissolving isobutyryl-l-cysteine (2 mg) and o-phthalaldehyde (1 mg) in 0.1 mL ethanol, followed by the addition of 0.9 mL sodium borate buffer (0.2 M, pH 9.0). Automated pre-column derivatives were obtained by drawing up a 5 μL aliquot of the standard or blank solution and 5 μL of the derivative reagent, and holding them in vials for 5 min before injection. The derivatized samples (5 μL) were injected by autosampler (xLC3059AS, Jasco). Analytical column (YMC Triat C18, particle 1.8 μm, 50 × 2.1 mm, YMC, Kyoto, Japan) was maintained at 45 °C and a flow rate of 500 μL/min. Linear gradient elution was performed over 10 min in mobile phases A (0.05 M citrate buffer, pH 5.0) and B (0.05 M citrate buffer containing 30% acetonitrile and 30% methanol, pH 3.5). The excitation/emission wavelengths of the fluorescence detector were set at 280 and 455 nm, respectively [46,47,48].

### 2.5. Simple Western Analysis

Simple Western analyses were performed using Wes (ProteinSimple, Santa Clara, CA) according to the ProteinSimple user manual [27]. Lysate of primary cultured astrocytes was mixed with a master mix (ProteinSimple) to a final concentration of 1×sample buffer, 1×fluorescent molecular weight markers, and 40 mM dithiothreitol, then heated at 95 °C for 5 min. The samples, blocking reagent, primary antibodies, horseradish peroxidase (HRP)-conjugated second antibodies, chemiluminescent substrate, and separation and stacking matrices were also dispensed to designated wells in a 25-well plate. After plate loading, the separation electrophoresis and immunodetection steps took place in the capillary system and were fully automated. Simple Western analysis was carried out at room temperature, and instrument default settings were used. Capillaries were first filled with separation matrix, followed by stacking matrix and about 40 nL sample loading. During electrophoresis, proteins were separated on the basis of molecular weight through the stacking and separation matrices at 250 volts for 40–50 min and then immobilized on the capillary wall using proprietary photo-activated capture chemistry. The matrices were then washed out. Capillaries were next incubated with a blocking reagent for 15 min, and target proteins were immunoprobed with primary antibodies, followed by HRP-conjugated secondary antibodies. Antibodies of GAPDH (NB300-322SS, Novus Biologicals, Littleton, CO) and Cx43 (C6219, Sigma-Aldrich, St. Louis, MO, USA) were diluted in antibody diluent (ProteinSimple) with 1:100 dilution. The antibody incubation time was 0–120 min with antibody diluents. Luminol and peroxide (ProteinSimple) were then added to generate chemiluminescence, which was captured by a charge coupled device (CCD) camera. The digital image was analyzed with Compass software (ProteinSimple), and the quantified data of the detected protein were reported as molecular weight, signal/peak intensity.

### 2.6. Statistical Analysis

All experiments were designed with equal-sized groups (*N* = 6) that were predetermined based on our previous studies [5,12,41,42,43,44]. All values are expressed as mean ± SD, and a *p* value less than 0.05 was considered statistically significant. Statistical analyses were performed in BellCurve for Excel Version 3.2. (Social Survey Research Information Co., Ltd., Tokyo, Japan). Astroglial transmitter concentrations were analyzed by Mauchly’s sphericity test, followed by multivariate analysis of variance (MANOVA) using BellCurve for Excel. When the data did not violate the assumption of sphericity (*p* > 0.05), the F-value of MANOVA was analyzed using sphericity-assumed degrees of freedom. However, if the assumption of sphericity was violated (*p* < 0.05), the F-value was analyzed using Chi-Muller’s-corrected degrees of freedom. When the F-value for the agent/concentration factors of MANOVA was significant, the data was analyzed by Tukey’s post hoc test. Protein expression of Cx43 in cytosol and plasma membrane fractions was analyzed by two-way analysis of variance (ANOVA) with Tukey’s post hoc test using BellCurve for Excel.

## 3. Results

### 3.1. Concentration-Dependent Effects of Acute Administration of CLZ on Astroglial Releases of L-glutamate and D-serine (Study_1)

**Figure 1:**
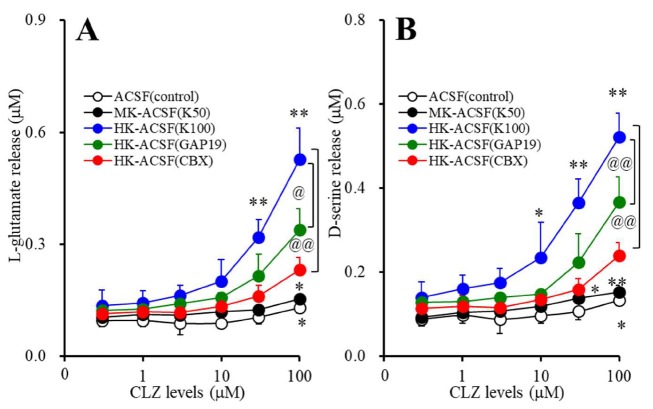
Concentration-dependent effects of acute administration of CLZ (clozapine) on basal and K^+^-evoked astroglial releases of l-glutamate (**A**) and d-serine (**B**). After wash-out, astrocytes were incubated in 100 μL ACSF (artificial cerebrospinal fluid) containing CLZ (0, 1, 3, 10, 30, or 100 μM) for 60 min (pretreatment incubation). After pretreatment incubation, to determine the K^+^-evoked astroglial releases of L-glutamate and D-serine, astrocytes were incubated in ACSF (3.0 mM K^+^: opened circles), MK-ACSF (50.0 mM K^+^: closed circles) or HK-ACSF (100.0 mM K^+^: blue circles) containing the same concentration of CLZ of pretreatment incubation for 20 min. To clarify the astroglial releases of L-glutamate and D-serine associated with Cxs (connexin (Cx) composed transmembrane channels), astrocytes were incubated in HK-ACSF containing GAP19 (TAT-conjugated Gap19, 20 μM) (green circles) or CBX (carbenoxolone, 100 μM) (red circles) with the same concentration of CLZ during pretreatment incubation for 20 min, and then incubation medium (ACSF, MK-ACSF or HK-ACSF) was collected for analysis. Ordinate: mean ± SD (*n* = 6) of extracellular levels of l-glutamate and d-serine (μM). Abscissa: concentration of CLZ (μM). * *p* < 0.05 and ** *p* < 0.01 vs. CLZ free by MANOVA with Tukey’s post hoc test. @ *p* < 0.05 and @@ *p* < 0.01 vs. HK-ACSF by MANOVA with Tukey’s post hoc test.

Acute administration of CLZ (0, 1, 3, 10, 30, and 100 μM) concentration-dependently increased basal and K^+^-evoked astroglial releases of L-glutamate (F_stimulation_(2,15) = 39.4 (*p* < 0.01), F_CLZ_(2.7,40.6) = 210.0 (*p* < 0.01), F_stimulation*CLZ_(5.4,40.6) = 114.4 (*P* < 0.01)) and D-serine (F_stimulation_(2,15) = 55.4 (*p* < 0.01), F_CLZ_(3.1,46.3) = 179.5 (*p* < 0.01), F_stimulation*CLZ_(6.2,46.3) = 86.7 (*p* < 0.01)) (Figure 1A,B). The K^+^-evoked stimulation (50 and 100 mM) concentration-dependently increased astroglial releases of L-glutamate and D-seine (Figure 1A,B). The threshold concentration of acute administration of CLZ on basal releases of L-glutamate and D-serine was 100 μM. The threshold concentration of acute administration of CLZ on 50 mM K^+^-evoked releases of L-glutamate and D-serine was 100 μM and 30 μM, respectively. The threshold concentration of acute administration of CLZ on 100 mM K^+^-evoked releases of L-glutamate and D-serine was 30 μM.

The selective Cx43 inhibitor, GAP19 (20 μM), decreased the CLZ-induced 100 mM K^+^-evoked astroglial releases of L-glutamate (F_GAP19_(1,10) = 8.5 (*p* < 0.01), F_CLZ_(2.6,25.9) = 259.1 (*p* < 0.01), F_GAP19*CLZ_ (2.6,25.9) = 22.6 (*p* < 0.01)) and D-serine (F_GAP19_(1,10) = 11.8 (*p* < 0.01), F_CLZ_(3.1,30.7) = 220.8 (*p* < 0.01), F_GAP19*CLZ_(3.1,30.7) = 13.8 (*p* < 0.01)) (Figure 1A,B). The nonselective Cxs inhibitor, CBX (100 μM), also decreased K^+^-evoked astroglial releases of L-glutamate (F_CBX_(1,10) = 20.6 (*p* < 0.01), F_CLZ_(2.2,22.1) = 226.1 (*p* < 0.01), F_CBX*CLZ_(2.2,22.1) = 66.3 (*p* < 0.01)) and D-serine (F_CBX_(1,10) = 26.5 (*p <* 0.01), F_CLZ_(3.3,32.8) = 178.1 (*p* < 0.01), F_CBX*CLZ_(3.3,32.8) = 48.1 (*p* < 0.01)) (Figure 1A,B). Therefore, both K^+^-evoked stimulation and acute administration of CLZ enhance astroglial releases of L-glutamate and D-serine associated with Cx43-based Cxs.

### 3.2. Concentration-Dependent Effects of Subchronic Administration of CLZ on Astroglial Releases of L-glutamate and D-serine (Study_2)

**Figure 2:**
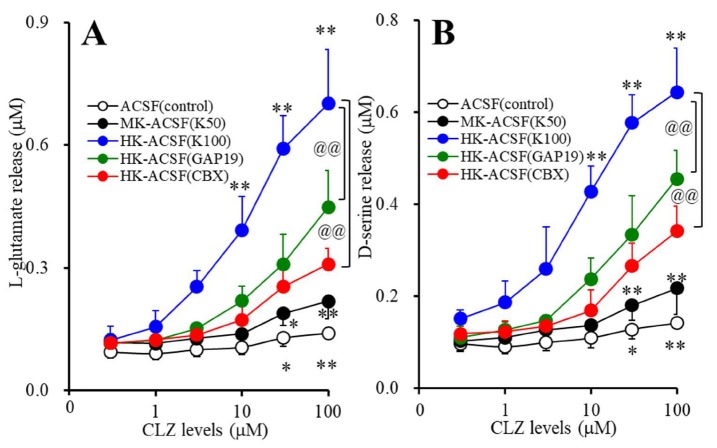
Concentration-dependent effects of subchronic administration of CLZ on basal and K^+^-evoked astroglial releases of L-glutamate (**A**) and D-serine (**B**). Astrocytes were incubated in Dulbecco’s modified Eagle’s medium containing 10% fetal calf serum (fDMEM) containing CLZ (0, 1, 3, 10, 30, or 100 μM) for 7 days. After wash-out, astrocytes were incubated in 100 μL ACSF containing the same concentration of CLZ for 60 min (pretreatment incubation). After pretreatment incubation, to determine the K^+^-evoked astroglial releases of L-glutamate and D-serine, astrocytes were incubated in ACSF (3.0 mM K^+^: opened circles), MK-ACSF (50.0 mM K^+^: closed circles), or HK-ACSF (100.0 mM K^+^: blue circles) containing the same concentration of CLZ during pretreatment incubation for 20 min. To clarify the astroglial releases of L-glutamate and D-serine associated with Cxs, astrocytes were incubated in HK-ACSF containing GAP19 (20 μM) (green circles) or CBX (100 μM) (red circles) with the same concentration of CLZ during pretreatment incubation for 20 min, and then incubate medium (ACSF, MK-ACSF, or HK-ACSF) was collected for analysis. Ordinate: mean ± SD (*n* = 6) of extracellular levels of l-glutamate and d-serine (μM). Abscissa: concentration of CLZ (μM). * *p* < 0.05 and ** *p* < 0.01 vs. CLZ free by MANOVA with Tukey’s post hoc test. @@ *p* < 0.01 vs. HK-ACSF by MANOVA with Tukey’s post hoc test.

Subchronic administration of CLZ (0, 1, 3, 10, 30, and 100 μM) concentration-dependently increased basal and K^+^-evoked astroglial releases of L-glutamate (F_stimulation_(2,15) = 67.1 (*p* < 0.01), F_CLZ_(1.5,22.5) = 268.1 (*p* < 0.01), F_stimulation*CLZ_(3.0,22.5) = 128.9 (*p* < 0.01)) and D-serine (F_stimulation_(2,15) = 80.6 (*p* < 0.01), F_CLZ_(2.2,32.3) = 287.8 (*p* < 0.01), F_stimulation*CLZ_(4.3,32.3) = 125.2 (*p* < 0.01)) (Figure 2A,B). The K^+^-evoked stimulation (50 and 100 mM) concentration-dependently increased astroglial releases of L-glutamate and D-seine (Figure 2A,B). The threshold concentration of subchronic administration of CLZ on basal releases of L-glutamate and D-serine was 30 μM. The threshold concentration of subchronic administration of CLZ on 50 mM K^+^-evoked releases of L-glutamate and D-serine was 30 μM. The threshold concentration of subchronic administration of CLZ on 100 mM K^+^-evoked releases of L-glutamate and D-serine was 10 μM.

The selective Cx43 inhibitor, GAP19 (20 μM), decreased the CLZ-induced 100 mM K^+^-evoked astroglial releases of L-glutamate (F_GAP19_(1,10) = 19.7 (*p* < 0.01), F_CLZ_(1.3,12.5) = 276.5 (*p* < 0.01), F_GAP19*CLZ_(1.3,12.5) = 27.0 (*p* < 0.01)) and D-serine (F_GAP19_(1,10) = 22.7 (*p* < 0.01), F_CLZ_(2.6,26.0) = 336.1 (*p* < 0.01), F_GAP19*CLZ_(2.6,26.0) = 18.6 (*p* < 0.01)) (Figure 2A,B). The nonselective Cxs inhibitor, CBX (100 μM), also decreased K^+^-evoked astroglial releases of L-glutamate (F_CBX_(1,10) = 35.6 (*p* < 0.01), F_CLZ_(1.6,15.6) = 279.7 (*p* < 0.01), F_CBX*CLZ_(1.6,15.6) = 72.0 (*p* < 0.01)) and D-serine (F_CBX_(1,10) = 38.3 (*p* < 0.01), F_CLZ_(2.0,20.2) = 296.3 (*p* < 0.01), FC_CBX__*CLZ_(2.0,20.2) = 52.0 (*p* < 0.01)) (Figure 2A,B). Therefore, both K^+^-evoked stimulation and acute administration of CLZ activate astroglial releases of L-glutamate and D-serine associated with Cx43-based Cxs.

### 3.3. Concentration-Dependent Effects of Acute and Subchronic Administrations of VPA on Astroglial Releases of L-glutamate and D-serine (Study_3)

**Figure 3:**
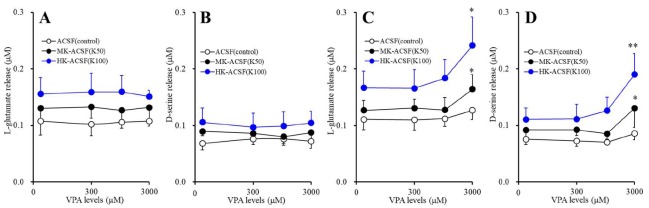
Concentration-dependent effects of acute administration of VPA (valproate) on basal and K^+^-evoked astroglial releases of l-glutamate (**A**) and d-serine (**B**). After wash-out, astrocytes were incubated in ACSF containing VPA (0, 300, 1000, or 3000 μM) for 60 min (pretreatment incubation). After pretreatment incubation, to determine the K^+^-evoked astroglial releases of L-glutamate and d-serine, astrocytes were incubated in ACSF (3.0 mM K^+^: opened circles), MK-ACSF (50.0 mM K^+^: closed circles), or HK-ACSF (100.0 mM K^+^: blue circles) containing the same concentration of VPA during pretreatment incubation for 20 min. Concentration-dependent effects of subchronic administration of VPA on basal and K^+^-evoked astroglial releases of l-glutamate (**C**) and d-serine (**D**). Astrocytes were incubated in fDMEM containing VPA (0, 300, 1000, or 3000 μM) for 7 days. After wash-out, astrocytes were incubated in ACSF containing the same concentration of VPA (pretreatment incubation). After pretreatment, to clarify the K^+^-evoked astroglial releases of l-glutamate and d-serine, astrocytes were incubated in ACSF (opened circles), MK-ACSF (closed circles), or HK-ACSF (blue circles) containing the same concentration of VPA during pretreatment incubation for 20 min, and then incubate medium (ACSF, MK-ACSF, or HK-ACSF) was collected for analysis. Ordinate: mean ± SD (*n* = 6) of extracellular levels of l-glutamate and d-serine (μM). Abscissa: concentration of VPA (μM). * *p* < 0.05 and ** *p* < 0.01 vs. VPA free by MANOVA with Tukey’s post hoc test.

Acute administration of VPA (0, 300, 1000, and 3000 μM) did not affect basal or K^+^-evoked astroglial releases of L-glutamate or D-serine (Figure 3A,B). Contrary to acute administration, subchronic administration of VPA (0, 300, 1000, and 3000 μM) concentration-dependently increased basal and K^+^-evoked astroglial releases of L-glutamate (F_stimulation_(2,15) = 13.6 (*p* < 0.01), F_VPA_(1.9,27.7) = 97.9 (*p* < 0.01), F_stimulation*VPA_(3.7,27.7) = 16.4 (*p* < 0.01)) and D-serine (F_stimulation_(2,15) = 16.5 (*p* < 0.01), F_VPA_(1.4,21.1) = 66.0 (*p* < 0.01), F_stimulation*VPA_(2.8,21.1) = 13.1 (*p* < 0.01)) (Figure 3C,D). Basal releases of L-glutamate and D-serine were not affected by VPA. Both 50 mM and 100 mM K^+^-evoked releases of L-glutamate and D-serine were increased by supratherapeutic concentration of VPA (3000 μM), but not affected by therapeutic-relevant concentration of VPA (300–1000 μM) (Figure 3C,D).

### 3.4. Acute Effects of Therapeutic-Relevant Concentration of VPA on Astroglial Releases of l-glutamate and d-serine from Astrocytes Subchronically Administrated with CLZ (Study_4)

**Figure 4:**
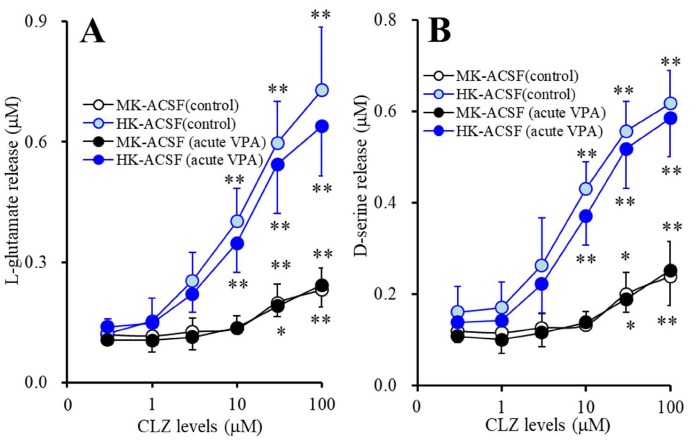
Acute effects of therapeutic-relevant concentration of VPA (1000 μM) on astroglial releases of L-glutamate (**A**) and D-serine (**B**) from astrocytes subchronically administrated with CLZ. Astrocytes were incubated in fDMEM containing CLZ (0, 1, 3, 10, 30, or 100 μM) for 7 days. After wash-out, astrocytes were incubated in ACSF containing the same concentration of CLZ without (control: opened circles) or with therapeutic-relevant concentration of VPA (1000 μM: closed circles) for 60 min (pretreatment incubation). After pretreatment, to determine the K^+^-evoked astroglial releases of L-glutamate and D-serine, astrocytes were incubated in MK-ACSF (50.0 mM K^+^: black circles) or HK-ACSF (100.0 mM K^+^: blue circles) containing the same concentrations of CLZ and VPA during pretreatment incubation for 20 min, and then incubate medium (MK-ACSF or HK-ACSF) was collected for analysis. Ordinate: mean ± SD (*n* = 6) of extracellular levels of l-glutamate and d-serine (μM). Abscissa: concentration of CLZ (μM). * *p* < 0.05 and ** *p* < 0.01 vs. CLZ free by MANOVA with Tukey’s post hoc test.

Subchronic administration of CLZ concentration-dependently increased K^+^-evoked astroglial releases of L-glutamate and D-serine (Figure 4A,B). Acute administration of therapeutic-relevant concentration of VPA (1000 μM) did not affect 50 mM K^+^-evoked releases of L-glutamate and D-serine from astrocytes subchronically administrated with CLZ (1–100 μM) (Figure 4A,B). Acute administration of therapeutic-relevant concentration of VPA (1000 μM) also did not affect 100 mM K^+^-evoked releases of L-glutamate and D-serine from astrocytes subchronically administrated with CLZ (1–100 μM) (Figure 4A,B).

### 3.5. Acute Effects of CLZ on Astroglial Releases of L-glutamate and D-serine from Astrocytes Subchronically Administrated with Therapeutic-Relevant Concentration of VPA (Study_5)

**Figure 5:**
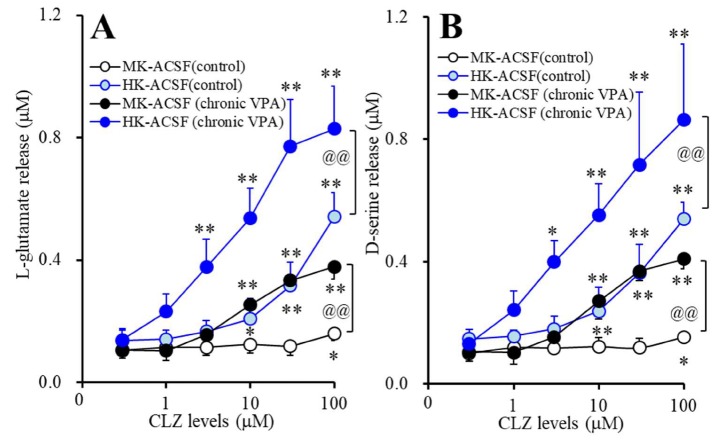
Acute effects of CLZ on astroglial releases of L-glutamate (**A**) and D-serine (**B**) from astrocytes subchronically administrated with therapeutic-relevant concentration of VPA (1000 μM). Astrocytes were incubated in fDMEM without (control: opened circles) or with VPA (1000 μM: closed circles). After wash-out, astrocytes were incubated in ACSF containing the same concentration of VPA with CLZ (0, 1, 3, 10, 30, 100 μM) for 60 min (pretreatment incubation). After pretreatment incubation, to determine the K^+^-evoked astroglial releases of L-glutamate and D-serine, astrocytes were incubated in MK-ACSF (50.0 mM K^+^: black circles) or HK-ACSF (100.0 mM K^+^: blue circles) containing the same concentration of CLZ and VPA during pretreatment incubation for 20 min, and then incubate medium (MK-ACSF or HK-ACSF) was collected for analysis. Ordinate: mean ± SD (*n* = 6) of extracellular levels of l-glutamate and d-serine (μM). Abscissa: concentration of CLZ (μM). * *p* < 0.05 and ** *p* < 0.01 vs. CLZ free, and @@ *p* < 0.01 vs. VPA free (control) by MANOVA with Tukey’s post hoc test.

Acute administration of CLZ concentration-dependently increased K^+^-evoked astroglial releases of L-glutamate and D-serine (Figure 5A,B). Subchronic administration of therapeutic-relevant concentration of VPA (1000 μM) increased astroglial CLZ-induced 50 mM K^+^-evoked releases of L-glutamate (F_VPA_(1,10) = 37.9 (*p* < 0.01), F_CLZ_(5,50) = 439.4 (*p* < 0.01), F_VPA*CLZ_(5,50) = 265.6 (*p* < 0.01)) and D-serine (F_VPA_(1,10) = 48.3 (*p* < 0.01), F_CLZ_(5,50) = 270.2 (*p* < 0.01), F_VPA*CLZ_(5,50) = 184.9 (*p* < 0.01)) (Figure 5A,B). Subchronic administration of therapeutic-relevant concentration of VPA (1000 μM) reduced the threshold concentration of acute administration of CLZ on 50 mM K^+^-evoked releases of L-glutamate (from 100 μM to 10 μM) and D-serine (from 30 μM to 10 μM).

Subchronic administration of therapeutic-relevant concentration of VPA (1000 μM) increased astroglial CLZ-induced 100 mM K^+^-evoked releases of L-glutamate (F_VPA_(1,10) = 30.1 (*p* < 0.01), F_CLZ_(1.9,19.1) = 291.8 (*p* < 0.01), F_VPA*CLZ_(1.9,19.1) = 40.3 (*p* < 0.01)) and D-serine (F_VPA_(1,10) = 16.3 (*p* < 0.01), F_CLZ_(1.6,15.9) = 94.7 (*p* < 0.01), F_VPA*CLZ_(1.6,25.9) = 11.5 (*p* < 0.01)) (Figure 5A,B). Subchronic VPA administration also reduced the threshold concentration of acute administration of CLZ on 100 mM K^+^-evoked releases of L-glutamate (from 30 μM to 3 μM) and D-serine (from 10 μM to 3 μM).

### 3.6. Interaction between VPA and CLZ on Cx43 Expression in Astrocytes (Studies_2,3,5)

**Figure 6:**
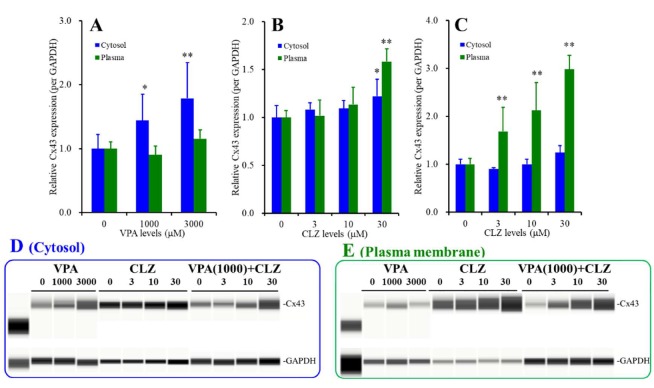
Subchronic effects of VPA (1000 and 3000 μM) (**A**) and CLZ (3, 10, and 30 μM) (**B**) on Cx43 expression in cytosol (blue columns) and plasma membrane (green columns) of astrocytes. Acute effects of CLZ (3, 10, and 30 μM) on Cx43 expression in cytosol and plasma membrane of subchronically treated astrocytes with therapeutic-relevant concentration of VPA (1000 μM) (**C**). Ordinate: mean ± SD (*n* = 6) of relative protein level of Cx43. Abscissa: concentration of VPA and CLZ (μM). * *p* < 0.05 and ** *p* < 0.01 vs. agent free by MANOVA with Tukey’s post hoc test. Panels 6(**D**) and 6(**E**) pseudo-gel images using Simple Western results using anti-GAPDH and anti-Cx43 antibody for blotting of cytosol and plasma membrane fractions, respectively.

Subchronic administrations of VPA (1000 and 3000 μM) concentration-dependently increased Cx43 expression in cytosol fraction without affecting that in plasma membrane fraction (F_Fragment_(1,30) = 7.4 (*p* < 0.01), F_Level_(2,30) = 15.0 (*p* < 0.01), F_Fragment*Level_(2,30) = 3.8 (*p* < 0.05)) (Figure 6A). Contrary to VPA, subchronic administrations of CLZ (3, 10, and 10 μM) concentration-dependently increased Cx43 expression in both cytosol and plasma membrane fractions (F_Fragment_(1,40) = 5.0 (*p* < 0.05), F_Level_(3,40) = 22.1 (*p* < 0.01), F_Fragment*Level_(3,40) = 6.2 (*p* < 0.01)) (Figure 6B). The threshold concentration of subchronic administration of CLZ on Cx43 expression in cytosol and plasma membrane was 30 μM, whereas the stimulatory effect of subchronic CLZ administration on Cx43 expression in plasma membrane was predominant, rather than that in cytosol (*p* < 0.05) (Figure 6B).

Acute administrations of CLZ (3, 10 and 10 μM) concentration-dependently increased Cx43 expression in plasma membrane but not cytosol fraction of astrocytes subchronically treated with therapeutic-relevant concentration of VPA (1000 μM) (F_Fragment_(1,40) = 33.4 (*p* < 0.01), F_Level_(3,40) = 15.6 (*p* < 0.01), F_Fragment*Level_(3,40) = 17.0 (*p* < 0.01)) (Figure 6C). The threshold concentration of acute administration of CLZ on Cx43 expression in plasma membrane of subchronically therapeutic-relevant concentration of VPA (1000 μM) treated astrocytes was 3 μM (*p* < 0.01) (Figure 6C).

## 4. Discussion

### 4.1. Effects of VPA and CLZ on Astroglial Transmission Associated with Cxs

The present study demonstrated that CLZ enhanced astroglial releases of L-glutamate and D-serine, in a concentration-dependent manner. Survival analysis has suggested that effective relapse prevention requires the maintenance of patients at CLZ serum concentrations above 0.6 μM [37]; however, exceeding 4 μM can significantly increase the risk of adverse effects such as seizures [38]. Therefore, acute and subchronic administrations of 1 and 3 μM CLZ are considered therapeutic-relevant range, whereas higher than 10 μM CLZ is considered supratherapeutic range. The threshold concentrations of acute administration of CLZ on basal, 50 mM and 100 mM K^+^-evoked astroglial releases of L-glutamate and D-serine were 100 μM, 30–100 μM and 10–30 μM, respectively. The K^+^-evoked astroglial release was composed of astroglial exocytosis [12,24,44], output through glutamate transporter [5,41,42], and Cxs [6]. Cxs activities were regulated by Ca^2+^, K^+^, and plasma membrane voltage [6,31]. During resting stage, Cxs exhibits a low open probability; however, the elevation of plasma membrane voltage activates Cxs [33]. In the present study, both nonselective Cxs inhibitor, CBX, and selective Cx43 inhibitor, GAP19, reduced the K^+^-evoked astroglial releases of L-glutamate and D-serine. These results suggest that the K^+^-evoked astroglial releases of L-glutamate and D-serine, at least partially, comprise the output through Cxs. Therefore, acute administration of CLZ concentration-dependently enhances astroglial releases of L-glutamate and D-serine associated with Cxs, but the stimulatory effects of CLZ on astroglial releases were observed in the supratherapeutic range. The therapeutic concentration of VPA was considered, ranged 350–700 μM [49]. Neither acute administration of therapeutic-relevant nor supratherapeutic concentrations of VPA (300–3000 μM) affected basal, 50 mM or 100 mM K^+^-evoked astroglial releases of L-glutamate and D-serine. Therefore, acute administration of VPA did not affect Cxs activities.

Contrary to acute administrations, chronic administration of CLZ also increased basal and K^+^-evoked astroglial releases of L-glutamate and D-serine. The stimulatory effects of subchronic administration of CLZ on basal and K^+^-evoked astroglial releases were predominant compared with those of acute CLZ administration, since the threshold concentrations of subchronic administration of CLZ on basal, 50 mM and 100 mM K^+^-evoked astroglial releases of L-glutamate and D-serine were reduced to 30 μM (acute: 100 μM), 30 μM (acute: 30–100 μM), and 10 μM (acute: 10–30 μM), respectively. Similar to CLZ, subchronic administration of supratherapeutic concentration of VPA (3000 μM) increased K^+^-evoked astroglial releases of L-glutamate and D-serine without affecting basal release. These discrepancies between acute and subchronic administrations of CLZ and VPA on basal and K^+^-evoked astroglial releases of L-glutamate and D-serine suggest that subchronic administration of CLZ and VPA possibly increases expression of Cxs in plasma membrane.

Indeed, simple western analysis detected the subchronic administration of CLZ and VPA increased astroglial expression of Cx43, which is the principal Cx isoform in astrocytes [50]; however, the expression patterns of Cx43 induced by subchronic administrations of VPA and CLZ are not identical. Subchronic administration of VPA increased Cx43 content in cytosol fraction concentration-dependently without affecting that in plasma membrane fraction. Contrary to subchronic administration of CLZ increased Cx43 content in both cytosol and plasma membrane fractions concentration-dependently, whereas an increase in expression of Cx43 in plasma membrane fraction was predominant, rather than those in cytosol fraction. Cx isoforms which are structural proteins in Cxs have an average half-life about 2–3 h [51]. Therefore, the increased Cx43 content induced by subchronic administration of VPA and CLZ is probably mediated by activation of Cx43 turnover.

The Cx gene regulation system, transcriptional factors (Sp1, activator protein 1 complex, cyclic adenosine monophosphate, Wnt signaling pathway), and epigenetic processes (histone modifications, DNA methylation and microRNA species) have been identified [31,52]. Histone deacetylase (HDAC) inhibition is considered to be one of the most principal pharmacological features of VPA, which inhibits Class I and IIa HDAC isoforms [53]. HDAC inhibitors are a class of drugs that increase the acetylation of histone and non-histone proteins to activate transcription, enhance gene expression, and modify the function of target proteins [53]. Both HDAC inhibitors, suberoylanilide hydroxamic acid and trichostatin A, increase expression of Cx43 mRNA and protein [54,55]. Additionally, subchronic administration of Class I and IIa HDAC inhibitor, 4-phenylbutyrate, for 5 days also increases Cx43 expression in NGC-407 cell [56]; however, two previous studies demonstrated VPA did not affect the astroglial expression of Cx43 [57,58]. Subacute (for 24 h) administration of VPA (300–1500 μM) did not affect total lysate fraction of Cx43 expression in astrocytes co-cultured with 5% or 30 % microglia [57]. The Cx43 expression in prefrontal cortex total lysate fraction of rat was not affected by chronically intraperitoneal administration of VPA (300 mg/kg/day for 21 days) [58]; however, the estimated concentration of VPA during subchronic administration of 300 mg/kg/day was lower than therapeutic-relevant range (about 300 μM) [49]. Therefore, taken together with these previous demonstrations, in spite of short half-life of Cx43 (about 2–3 h) [51], the present results suggest that the elevation of Cx43 expression in cytosol fraction of astrocytes induced by VPA needs more than 5 days exposure with higher than therapeutic-relevant concentration of VPA (1000 μM).

Contrary to VPA, CLZ activates the inhibitory and stimulatory expression regulating system on Cx43 synthesis, such as HDAC2, Wnt pathway, transcription factor specificity protein 1, and activator protein 1 complex [59,60,61,62]. The effect of CLZ on Cx43 expression has more impact against post-translational modification than gene expression, since the CLZ-induced Cx43 expression in plasma membrane fraction was higher than that in the cytosol fraction. The post-translational modification of Cx43, including phosphorylation, acetylation, nitrosylation, sumoylation, and ubiquitylation, plays important roles in the Cxs function [31]. Especially, inhibition of ubiquitylation of Cx43 results in the accumulation of Cx43 in plasma membrane [63]. Unfortunately, the effects of CLZ on phosphorylation, acetylation, nitrosylation, sumoylation, and ubiquitylation systems have not been clarified. The detailed mechanism of an increase in the Cx43 expression in the astroglial plasma membrane shall be studied.

Cx43 is the principal Cxs in astrocytes, but Cx26 and Cx30 are also expressed in astrocytes [30]. In spite of lower quantities of Cx26 and Cx30, the hemichannel composed of Cx26 and Cx30 also contributes to astroglial transmission [30]. In the present study, the inhibitory effects of nonselective Cxs inhibitor, CBX, on astroglial releases of L-glutamate and D-serine were predominant compared with selective Cx43 inhibitor, GAP19. Therefore, to clarify the mechanisms of CLZ and VPA on astroglial transmission associated with Cxs, further study should determine the effects of them on astroglial transmission associated with Cx26 and Cx30.

### 4.2. Interaction between VPA and CLZ

The incidence of seizures with CLZ is ranged from 2% to 7.5%, and the risk of CLZ-induced seizures increased with higher doses [18]. The risk of CLZ-induced seizure was increased dose-dependently: lower than 300 mg/day (3%), between 325 and 500 mg/day (8%), and higher than 500 mg/day (38%) [18]. In 50% cases of CLZ-induced seizures, reduced CLZ dose was sufficient to prevent recurrence of seizures [18]. VPA is recommended as the first-line drug in prevention of CLZ-induced seizure and augmentation therapy [20,21]. In the present study, acute administration of therapeutic-relevant concentration of VPA did not affect astroglial releases of L-glutamate and D-serine from astrocytes subchronically administrated with CLZ. Contrary to our expectations, the present study failed to detect the specific novel mechanism of VPA on astroglial transmission of chronically CLZ-exposed astrocytes. Most of the cognitive and behavioral functions associated with astroglial Cx43 have been implicated in the ability to regulate astroglial transmitter release through Cxs during resting stage [64]. Therefore, the present demonstration, the lack of acute VPA administration on astroglial transmission of chronically CLZ-exposed astrocytes, supports the mechanisms of efficacious and safe augmentation agents of CLZ due to less cognitive impairment induced by Cxs [20].

In the present study, neither acute nor subchronic administrations of therapeutic-relevant concentration of CLZ (1–3 μM) affected astroglial transmission; however, acute administration of therapeutic-relevant concentration of CLZ drastically increased astroglial releases of L-glutamate and D-serine from astrocytes which were chronically administrated with therapeutic-relevant concentration of VPA (1000 μM). Therapeutic-relevant concentration of CLZ (3 μM) also drastically increased Cx43 expression in the plasma membrane fraction without affecting that in cytosol fraction of therapeutic-relevant concentration of VPA-administrated astrocytes. These results suggest that increased Cx43 in the cytosol fraction induced by subchronic administration of therapeutic-relevant concentration of VPA (1000 μM) is transported to plasma membrane by CLZ. The rapid titration of CLZ dose and VPA administration at the commencing CLZ are significantly associated with increasing risks of CLZ-induced myocarditis/cardiomyopathies [22]. It has been well established that chronic downregulation of Cx43 is observed in several models of myocarditis and cardiomyopathies [65]. In contrast with the above observation of Cx43 downregulation, expression of Cx43 was increased in the early stage of hypertrophic and dilated cardiomyopathies, but with their progression into heart failure, the levels of Cx43 decreased [65]. Therefore, upregulation of Cx43 in acute stages of hypertrophic and dilated cardiomyopathies may act as the trigger of pathological or pathophysiological onset. Moreover, prolongation of QRS complex duration is one of the risk factors for morbidity and mortality in myocarditis. Recent preclinical study demonstrated that upregulation of Cx43 contributed to the prolongation of QRS complex duration [33]. Taken together with previous clinical and preclinical findings, the present demonstration suggests that the upregulation of cardiac Cx43 in cytosol by chronic administration of therapeutic-relevant concentration of VPA with enhancement of transported cytosol Cx43 to plasma membrane induced by CLZ plays an important role in the pathomechanisms of CLZ-induced myocarditis/cardiomyopathies. To clarify our hypothesis, further studies are needed.

## 5. Conclusions

The present study determined the concentration- and time-dependent effects of CLZ and VPA on astroglial transmission of L-glutamate and D-serine associated with Cx43, to explore the mechanisms of the multimodal action of CLZ. Both acute and subchronic administrations of CLZ and VPA increased astroglial releases of L-glutamate and D-serine concentration-dependently, but the therapeutic-relevant concentration of neither CLZ nor VPA affected these releases. The stimulatory effects of subchronic administrations of CLZ and VPA on astroglial releases were more predominant compared with those of acute administrations. Subchronic administrations of both VPA and CLZ concentration-dependently increased Cx43 expression in astrocytes, but the therapeutic-relevant concentration of neither CLZ nor VPA affected Cx43 expression. Especially, VPA increased Cx43 expression in cytosol fraction of astrocytes, whereas CLZ increased Cx43 expression in both cytosol and plasma membrane fractions. After subchronic administration of CLZ, acute administration of therapeutic-relevant concentration of VPA did not affect CLZ-induced astroglial transmitter releases; however, after subchronic administration of therapeutic-relevant concentration of VPA, acute administration of CLZ drastically increased astroglial transmitter releases. Therapeutic-relevant concentration of CLZ alone could not affect astroglial release, whereas after the subchronic administration of therapeutic-relevant concentration of VPA, acute administration of therapeutic-relevant concentration of CLZ could increase astroglial transmitter releases. During subchronic administration of therapeutic-relevant concentration of VPA, acute administration of therapeutic-relevant concentration of CLZ enhanced the transport of cytosol Cx43 to plasma membrane. Therefore, CLZ enhances astroglial functional Cxs via activation of transport of cytosol Cx43 to plasma membrane. These results suggest that the hyperactivation of astroglial Cxs activities induced by supratherapeutic concentration or rapid titration of CLZ, at least partially, contributes to CLZ-induced seizure, but inhibitory effects of VPA on CLZ-induced seizure are not modulated with astroglial transmission associated with Cxs. Interestingly, VPA intake at the commencing CLZ increases risk of CLZ-induced myocarditis/cardiomyopathies, probably via reciprocal activation of Cxs between VPA and CLZ.

## References

[1] Lieberman J.A., Bymaster F.P., Meltzer H.Y., Deutch A.Y., Duncan G.E., Marx C.E., Aprille J.R., Dwyer D.S., Li X.M., Mahadik S.P. (2008). Antipsychotic drugs: Comparison in animal models of efficacy, neurotransmitter regulation, and neuroprotection. Pharmacol. Rev..

[2] Meltzer H.Y., Huang M. (2008). In vivo actions of atypical antipsychotic drug on serotonergic and dopaminergic systems. Prog. Brain Res..

[3] Okada M., Fukuyama K., Ueda Y. (2019). Lurasidone inhibits NMDA receptor antagonist-induced functional abnormality of thalamocortical glutamatergic transmission via 5-HT7 receptor blockade. Br. J. Pharmacol..

[4] Okada M., Fukuyama K., Okubo R., Shiroyama T., Ueda Y. (2019). Lurasidone Sub-Chronically Activates Serotonergic Transmission via Desensitization of 5-HT1A and 5-HT7 Receptors in Dorsal Raphe Nucleus. Pharmaceuticals.

[5] Okada M., Fukuyama K., Kawano Y., Shiroyama T., Ueda Y. (2019). Memantine protects thalamocortical hyper-glutamatergic transmission induced by NMDA receptor antagonism via activation of system xc. Pharmacol. Res. Perspect..

[6] Fukuyama K., Kato R., Murata M., Shiroyama T., Okada M. (2019). Clozapine Normalizes a Glutamatergic Transmission Abnormality Induced by an Impaired NMDA Receptor in the Thalamocortical Pathway via the Activation of a Group III Metabotropic Glutamate Receptor. Biomolecules.

[7] Fukuyama K., Hasegawa T., Okada M. (2018). Cystine/Glutamate Antiporter and Aripiprazole Compensate NMDA Antagonist-Induced Dysfunction of Thalamocortical L-Glutamatergic Transmission. Int. J. Mol. Sci..

[8] Mitterauer B. (2009). Loss of function of glial gap junctions may cause severe cognitive impairments in schizophrenia. Med. Hypotheses.

[9] Okada M., Fukuyama K., Nakano T., Ueda Y. (2019). Pharmacological Discrimination of Effects of MK801 on Thalamocortical, Mesothalamic, and Mesocortical Transmissions. Biomolecules.

[10] Javitt D.C. (2007). Glutamate and schizophrenia: Phencyclidine, N-methyl-D-aspartate receptors, and dopamine-glutamate interactions. Int. Rev. Neurobiol..

[11] Labrie V., Roder J.C. (2010). The involvement of the NMDA receptor D-serine/glycine site in the pathophysiology and treatment of schizophrenia. Neurosci. Biobehav. Rev..

[12] Fukuyama K., Okada M. (2018). Effects of levetiracetam on astroglial release of kynurenine-pathway metabolites. Br. J. Pharmacol..

[13] Malhotra A.K., Pinals D.A., Weingartner H., Sirocco K., Missar C.D., Pickar D., Breier A. (1996). NMDA receptor function and human cognition: The effects of ketamine in healthy volunteers. Neuropsychopharmacology.

[14] Krystal J.H., Karper L.P., Seibyl J.P., Freeman G.K., Delaney R., Bremner J.D., Heninger G.R., Bowers M.B., Charney D.S. (1994). Subanesthetic effects of the noncompetitive NMDA antagonist, ketamine, in humans. Psychotomimetic, perceptual, cognitive, and neuroendocrine responses. Arch. Gen. Psychiatry.

[15] Malhotra A.K., Pinals D.A., Adler C.M., Elman I., Clifton A., Pickar D., Breier A. (1997). Ketamine-induced exacerbation of psychotic symptoms and cognitive impairment in neuroleptic-free schizophrenics. Neuropsychopharmacology.

[16] Krystal J.H., D’Souza D.C., Mathalon D., Perry E., Belger A., Hoffman R. (2003). NMDA receptor antagonist effects, cortical glutamatergic function, and schizophrenia: Toward a paradigm shift in medication development. Psychopharmacology.

[17] Farooq S., Choudry A., Cohen D., Naeem F., Ayub M. (2019). Barriers to using clozapine in treatment-resistant schizophrenia: Systematic review. BJPsych Bull..

[18] Grover S., Hazari N., Chakrabarti S., Avasthi A. (2015). Association of Clozapine with Seizures: A Brief Report Involving 222 Patients Prescribed Clozapine. East Asian Arch. Psychiatry.

[19] Farooq S., Taylor M. (2011). Clozapine: Dangerous orphan or neglected friend?. Br. J. Psychiatry.

[20] Zheng W., Xiang Y.T., Yang X.H., Xiang Y.Q., de Leon J. (2017). Clozapine Augmentation With Antiepileptic Drugs for Treatment-Resistant Schizophrenia: A Meta-Analysis of Randomized Controlled Trials. J. Clin. Psychiatry.

[21] David M., Taylor T.R.E.B., Young A.H. (2018). The Maudsley Prescribing Guidelines in Psychiatry.

[22] Ronaldson K.J., Fitzgerald P.B., Taylor A.J., Topliss D.J., Wolfe R., McNeil J.J. (2012). Rapid clozapine dose titration and concomitant sodium valproate increase the risk of myocarditis with clozapine: A case-control study. Schizophrenia Res..

[23] Siskind D., McCartney L., Goldschlager R., Kisely S. (2016). Clozapine v. first- and second-generation antipsychotics in treatment-refractory schizophrenia: Systematic review and meta-analysis. Br. J. Psychiatry.

[24] Tanahashi S., Yamamura S., Nakagawa M., Motomura E., Okada M. (2012). Clozapine, but not haloperidol, enhances glial D-serine and L-glutamate release in rat frontal cortex and primary cultured astrocytes. Br. J. Pharmacol..

[25] Malhotra A.K., Adler C.M., Kennison S.D., Elman I., Pickar D., Breier A. (1997). Clozapine blunts N-methyl-D-aspartate antagonist-induced psychosis: A study with ketamine. Biol. Psychiatry.

[26] Okada M., Fukuyama K., Kawano Y., Shiroyama T., Suzuki D., Ueda Y. (2019). Effects of acute and sub-chronic administrations of guanfacine on catecholaminergic transmissions in the orbitofrontal cortex. Neuropharmacology.

[27] Fukuyama K., Fukuzawa M., Shiroyama T., Okada M. (2020). Pathogenesis and pathophysiology of autosomal dominant sleep-related hypermotor epilepsy with S284L-mutant alpha4 subunit of nicotinic ACh receptor. Br. J. Pharmacol..

[28] Lapato A.S., Tiwari-Woodruff S.K. (2018). Connexins and pannexins: At the junction of neuro-glial homeostasis & disease. J. Neurosci. Res..

[29] Li Q., Li Q.Q., Jia J.N., Liu Z.Q., Zhou H.H., Mao X.Y. (2019). Targeting gap junction in epilepsy: Perspectives and challenges. Biomed. Pharmacother..

[30] Medina-Ceja L., Salazar-Sanchez J.C., Ortega-Ibarra J., Morales-Villagran A. (2019). Connexins-Based Hemichannels/Channels and Their Relationship with Inflammation, Seizures and Epilepsy. Int. J. Mol. Sci..

[31] Ribeiro-Rodrigues T.M., Martins-Marques T., Morel S., Kwak B.R., Girao H. (2017). Role of connexin 43 in different forms of intercellular communication—Gap junctions, extracellular vesicles and tunnelling nanotubes. J. Cell Sci..

[32] Dallerac G., Rouach N. (2016). Astrocytes as new targets to improve cognitive functions. Prog. Neurobiol..

[33] Zhong C., Chang H., Wu Y., Zhou L., Wang Y., Wang M., Wu P., Qi Z., Zou J. (2018). Up-regulated Cx43 phosphorylation at Ser368 prolongs QRS duration in myocarditis. J. Cell. Mol. Med..

[34] Mylvaganam S., Ramani M., Krawczyk M., Carlen P.L. (2014). Roles of gap junctions, connexins, and pannexins in epilepsy. Front. Physiol..

[35] Wu X.M., Wang G.L., Miao J., Feng J.C. (2015). Effect of connexin 36 blockers on the neuronal cytoskeleton and synaptic plasticity in kainic acid-kindled rats. Transl. Neurosci..

[36] Jin M., Dai Y., Xu C., Wang Y., Wang S., Chen Z. (2013). Effects of meclofenamic acid on limbic epileptogenesis in mice kindling models. Neurosci. Lett..

[37] Xiang Y.Q., Zhang Z.J., Weng Y.Z., Zhai Y.M., Li W.B., Cai Z.J., Tan Q.R., Wang C.Y. (2006). Serum concentrations of clozapine and norclozapine in the prediction of relapse of patients with schizophrenia. Schizophrenia Res..

[38] Varma S., Bishara D., Besag F.M., Taylor D. (2011). Clozapine-related EEG changes and seizures: Dose and plasma-level relationships. Ther. Adv. Psychopharmacol..

[39] Wang N., De Bock M., Decrock E., Bol M., Gadicherla A., Bultynck G., Leybaert L. (2013). Connexin targeting peptides as inhibitors of voltage- and intracellular Ca2+-triggered Cx43 hemichannel opening. Neuropharmacology.

[40] McGrath J.C., Drummond G.B., McLachlan E.M., Kilkenny C., Wainwright C.L. (2010). Guidelines for reporting experiments involving animals: The ARRIVE guidelines. Br. J. Pharmacol..

[41] Okada M., Fukuyama K., Shiroyama T., Ueda Y. (2019). Carbamazepine Attenuates Astroglial L-Glutamate Release Induced by Pro-Inflammatory Cytokines via Chronically Activation of Adenosine A2A Receptor. Int. J. Mol. Sci..

[42] Nakano T., Hasegawa T., Suzuki D., Motomura E., Okada M. (2019). Amantadine Combines Astroglial System Xc(-) Activation with Glutamate/NMDA Receptor Inhibition. Biomolecules.

[43] Fukuyama K., Tanahashi S., Hoshikawa M., Shinagawa R., Okada M. (2014). Zonisamide regulates basal ganglia transmission via astroglial kynurenine pathway. Neuropharmacology.

[44] Yamamura S., Hoshikawa M., Kato D., Saito H., Suzuki N., Niwa O., Okada M. (2013). ONO-2506 inhibits spike-wave discharges in a genetic animal model without affecting traditional convulsive tests via gliotransmission regulation. Br. J. Pharmacol..

[45] Tanahashi S., Yamamura S., Nakagawa M., Motomura E., Okada M. (2012). Dopamine D2 and serotonin 5-HT1A receptors mediate the actions of aripiprazole in mesocortical and mesoaccumbens transmission. Neuropharmacology.

[46] Yamamura S., Ohoyama K., Hamaguchi T., Nakagawa M., Suzuki D., Matsumoto T., Motomura E., Tanii H., Shiroyama T., Okada M. (2009). Effects of zotepine on extracellular levels of monoamine, GABA and glutamate in rat prefrontal cortex. Br. J. Pharmacol..

[47] Yoshida S., Okada M., Zhu G., Kaneko S. (2007). Carbamazepine prevents breakdown of neurotransmitter release induced by hyperactivation of ryanodine receptor. Neuropharmacology.

[48] Okada M., Yoshida S., Zhu G., Hirose S., Kaneko S. (2005). Biphasic actions of topiramate on monoamine exocytosis associated with both soluble N-ethylmaleimide-sensitive factor attachment protein receptors and Ca(2+)-induced Ca(2+)-releasing systems. Neuroscience.

[49] Yoshida S., Yamamura S., Ohoyama K., Nakagawa M., Motomura E., Kaneko S., Okada M. (2010). Effects of valproate on neurotransmission associated with ryanodine receptors. Neurosci. Res..

[50] Zancan M., Malysz T., Moura D.J., Moras A.M., Steffens L., Rasia-Filho A.A. (2019). Gap junctions and expression of Cx36, Cx43 and Cx45 in the posterodorsal medial amygdala of adult rats. Histol. Histopathol..

[51] Flores C.E., Nannapaneni S., Davidson K.G., Yasumura T., Bennett M.V., Rash J.E., Pereda A.E. (2012). Trafficking of gap junction channels at a vertebrate electrical synapse in vivo. Proc. Natl. Acad. Sci. USA.

[52] Oyamada M., Takebe K., Oyamada Y. (2013). Regulation of connexin expression by transcription factors and epigenetic mechanisms. Biochim. Biophys. Acta.

[53] Fessler E.B., Chibane F.L., Wang Z., Chuang D.M. (2013). Potential roles of HDAC inhibitors in mitigating ischemia-induced brain damage and facilitating endogenous regeneration and recovery. Curr. Pharm. Des..

[54] Hernandez M., Shao Q., Yang X.J., Luh S.P., Kandouz M., Batist G., Laird D.W., Alaoui-Jamali M.A. (2006). A histone deacetylation-dependent mechanism for transcriptional repression of the gap junction gene cx43 in prostate cancer cells. Prostate.

[55] Ogawa T., Hayashi T., Tokunou M., Nakachi K., Trosko J.E., Chang C.C., Yorioka N. (2005). Suberoylanilide hydroxamic acid enhances gap junctional intercellular communication via acetylation of histone containing connexin 43 gene locus. Cancer Res..

[56] Khan Z., Akhtar M., Asklund T., Juliusson B., Almqvist P.M., Ekstrom T.J. (2007). HDAC inhibition amplifies gap junction communication in neural progenitors: Potential for cell-mediated enzyme prodrug therapy. Exp. Cell Res..

[57] Dambach H., Hinkerohe D., Prochnow N., Stienen M.N., Moinfar Z., Haase C.G., Hufnagel A., Faustmann P.M. (2014). Glia and epilepsy: Experimental investigation of antiepileptic drugs in an astroglia/microglia co-culture model of inflammation. Epilepsia.

[58] Fatemi S.H., Folsom T.D., Reutiman T.J., Pandian T., Braun N.N., Haug K. (2008). Chronic psychotropic drug treatment causes differential expression of connexin 43 and GFAP in frontal cortex of rats. Schizophrenia Res..

[59] de la Fuente Revenga M., Ibi D., Cuddy T., Toneatti R., Kurita M., Ijaz M.K., Miles M.F., Wolstenholme J.T., Gonzalez-Maeso J. (2019). Chronic clozapine treatment restrains via HDAC2 the performance of mGlu2 receptor agonism in a rodent model of antipsychotic activity. Neuropsychopharmacology.

[60] Sutton L.P., Honardoust D., Mouyal J., Rajakumar N., Rushlow W.J. (2007). Activation of the canonical Wnt pathway by the antipsychotics haloperidol and clozapine involves dishevelled-3. J. Neurochem..

[61] Pinacho R., Valdizan E.M., Pilar-Cuellar F., Prades R., Tarrago T., Haro J.M., Ferrer I., Ramos B. (2014). Increased SP4 and SP1 transcription factor expression in the postmortem hippocampus of chronic schizophrenia. J. Psychiatr. Res..

[62] Kontkanen O., Lakso M., Wong G., Castren E. (2002). Chronic antipsychotic drug treatment induces long-lasting expression of fos and jun family genes and activator protein 1 complex in the rat prefrontal cortex. Neuropsychopharmacology.

[63] Girao H., Catarino S., Pereira P. (2009). Eps15 interacts with ubiquitinated Cx43 and mediates its internalization. Exp. Cell Res..

[64] Chever O., Lee C.Y., Rouach N. (2014). Astroglial connexin43 hemichannels tune basal excitatory synaptic transmission. J. Neurosci..

[65] Fontes M.S., van Veen T.A., de Bakker J.M., van Rijen H.V. (2012). Functional consequences of abnormal Cx43 expression in the heart. Biochim. Biophys. Acta.

